# Optimizing Epoxy Molding Compound Processing: A Multi-Sensor Approach to Enhance Material Characterization and Process Reliability

**DOI:** 10.3390/polym16111540

**Published:** 2024-05-30

**Authors:** Julian Vogelwaid, Martin Bayer, Michael Walz, Felix Hampel, Larysa Kutuzova, Günter Lorenz, Andreas Kandelbauer, Timo Jacob

**Affiliations:** 1 Mobility Electronics, Engineering Technology Polymer & Packaging, Robert Bosch GmbH, 72770 Reutlingen, Germany; martin.bayer2@de.bosch.com (M.B.); michaeldavid.walz@de.bosch.com (M.W.); fixed-term.felix.hampel@de.bosch.com (F.H.); 2 Fakultät für Naturwissenschaften, Institut für Elektrochemie, Universität Ulm, 89081 Ulm, Germany; timo.jacob@uni-ulm.de; 3 Fakultät für Life Sciences, Reutlingen University, 72762 Reutlingen, Germany; larysa.kutuzova@reutlingen-university.de (L.K.); guenter.lorenz@reutlingen-university.de (G.L.); andreas.kandelbauer@reutlingen-university.de (A.K.); 4 Department of Material Sciences and Process Engineering, Institute of Wood Technology and Renewable Materials, University of Natural Resources and Life Sciences, 1180 Vienna, Austria

**Keywords:** dielectric analysis (DEA), epoxy molding compound (EMC), monitoring, dynamic mechanical analysis (DMA), glass transition temperature (*T*_g_), response surface, heterogeneity

## Abstract

The in-line control of curing during the molding process significantly improves product quality and ensures the reliability of packaging materials with the required thermo-mechanical and adhesion properties. The choice of the morphological and thermo-mechanical properties of the molded material, and the accuracy of their determination through carefully selected thermo-analytical methods, play a crucial role in the qualitative prediction of trends in packaging product properties as process parameters are varied. This work aimed to verify the quality of the models and their validation using a highly filled molding resin with an identical chemical composition but 10 wt% difference in silica particles (SPs). Morphological and mechanical material properties were determined by dielectric analysis (DEA), differential scanning calorimetry (DSC), warpage analysis and dynamic mechanical analysis (DMA). The effects of temperature and injection speed on the morphological properties were analyzed through the design of experiments (DoE) and illustrated by response surface plots. A comprehensive approach to monitor the evolution of ionic viscosity (IV), residual enthalpy (*d*H_rest_), glass transition temperature (*T_g_*), and storage modulus (E) as a function of the transfer-mold process parameters and post-mold-cure (PMC) conditions of the material was established. The reliability of *T_g_* estimation was tested using two methods: warpage analysis and DMA. The noticeable deterioration in the quality of the analytical signal for highly filled materials at high cure rates is discussed. Controlling the temperature by increasing the injection speed leads to the formation of a polymer network with a lower *T_g_* and an increased storage modulus, indicating a lower density and a more heterogeneous structure due to the high heating rate and shear heating effect.

## 1. Introduction

Epoxy molding compounds (EMCs) are thermosets with excellent packaging properties for the encapsulation of integrated circuit boards, hybrid circuit boards, and transistors in the electronics and microelectronics industries for semiconductor devices and microchips [1,2]. Primary processing methods for EMCs include injection molding, compression molding and transfer molding [1,2,3,4].

EMCs frequently contain significant amounts of micro-size silica particles (SPs) to increase the stiffness and strength of the epoxy-molded products. SPs improve thermal and electrical insulation [5,6,7] and increase the storage modulus, which results in a reduction in the elasticity of the EMC [6]. The desired durability and mechanical characteristics are achieved by careful selection, optimal proportions and correct integration [8]. On the other hand, depending on the interaction between the polymer and the filler, the reaction can be influenced by the integration of fillers into the thermoset’s composition [9]. The surface of these fillers can be covered with functional silane, which functions as an adhesion promoter to improve the bonding between the filler and matrix. The result is an increase in reaction rate with increasing filler content through the catalysis of the epoxide–amine reaction and interfacial effects [10,11]. Conversely, the filler particle size affects the reaction rate. Decreasing the filler particle size causes the activation energy to decrease, even at 0.1 wt% filler [12]. In addition, it has been shown that a reduced silica content in the molding material can accelerate the reaction and thus the development of morphological properties such as degree of cure and *T_g_* [13]. The higher thermal conductivity and the increased organic content in the material contribute significantly to the accelerated reaction [13,14].

Over the last decade, process control has evolved and become an increasingly important part of the molding process. As a result, the number of different sensors integrated into the cavities of molding tools has significantly increased to ensure process stability [3].

Process stability in terms of cavity filling and reaction temperature can be controlled by monitoring temperature and pressure. Furthermore, understanding the curing behavior of thermosets during the process, during which morphological properties, e.g., *T*_g_ are changing, is essential for achieving suitable molding results. Implementing a sensor system to monitor the cross-linking process is therefore an essential element of process optimization.

Process optimization and ensuring consistently high product quality is a major challenge in industrial practice. Real-time information on the curing state of the material during reaction cannot be determined by simple temperature and pressure sensors [15]. Offline methods, e.g., differential scanning calorimetry (DSC), dynamic–mechanical analysis (DMA) or thermo-mechanical analysis (TMA) are commonly used to determine morphological properties such as the degree of cure and the *T*_g_. However, the curing conditions simulated during these measurements are not directly comparable to molding processes like the transfer-mold process [15,16,17,18,19]. Alternatively, on-line monitoring sensors such as dielectric analysis (DEA), infrared (IR) spectroscopy, Raman spectroscopy, and ultrasonic monitoring can be used to determine the kinetics of polymers in real-time [19,20,21].

DEA has enormous potential for industrial use as a process control tool due to its higher robustness compared to other sensor technologies, easier integration into tooling and, most importantly, lower costs [22]. Furthermore, DEA is capable of measuring opaque materials, which provides it with an advantage over optical techniques [21,22,23]. However, compared to spectroscopic and ultrasonic cure monitoring tools, DEA is sensitive to temperature impacts, inclusive of shearing. [24]. In addition, during the melting phase and solidification phase, spectroscopic methods are more accurate [25]. The temperature dependence of the DEA derives from the thermodynamics of the ion mobility at an induced temperature. This decreases with increasing temperature, as the ion mobility is restricted to a maximum, comparable to a limiting exponential behavior [26,27,28]. The temperature dependance can be removed by applying an empirical factor [24]. A correlation between the ionic viscosity (IV) and the degree of curing has been demonstrated in a number of studies [22,29,30,31,32]. Moreover, a linear correlation between IV and *T*_g_ through the implementation of a temperature compensation factor was demonstrated [24]. On this basis, a direct prediction of *T*_g_ using IV was demonstrated, assuming that IV behaves in a comparable way to the *T*_g_ via DiBenedetto [33].

The cure of thermosets is characterized by two separate phenomena: gelation and vitrification [34]. Gelation represents the beginning of the formation of a polymer matrix with an unlimited average molecular weight, while vitrification is linked to the transition from the visco-elastic state to the glassy state. 

Most thermosets used in industry are thermally activated systems and are processed via injection molding or transfer molding [23,24,25,26,27,28,29,30,31,32,33,34,35]. In these cases, knowledge of the gelation and vitrification times of the curing system at each temperature is essential to create an appropriate cure schedule for a specific application.

Since the initiation of the reaction is temperature-dependent, process parameters such as injection speed and temperature are critical for the formation of the polymer network. As a consequence, mechanical and thermo-mechanical properties can be influenced [36,37,38,39,40,41,42,43,44]. In particular, the heteropolymerization of thermosets, which leads to an inhomogeneous or heterogeneous network structure, has been extensively discussed in the literature. In general, it can be assumed that all polymer networks of thermosets are heterogeneous [35,36,37]. Heterogeneous networks may be formed by the following:

▪ Reaction mechanisms that favor the formation of regions of higher and lower cross-linking density, e.g., multiple microgels or reaction centers.
▪ Formation of chemical clusters of different natures.
▪ Changes in thermodynamic interactions (number and type of interaction sites).
▪ Increasing molecular weights (before the gel point).
▪ Increasing cross-linking density (after the gel point).
▪ Presence of diluents or additives.
▪ Curing below the final *T*_g1_ [36,37,41,45].

The aforementioned causes for the increase in heterogeneity vary greatly depending on the cross-linking system; however, these effects are primarily influenced by temperature-influencing process parameters such as temperature and injection speed [35,36,43]. The heterogeneity of thermosets, such as epoxy–phenolic systems, is difficult to see and has only been detected using atomic force microscopy and infrared spectroscopy [46,47]. The effects of heterogeneities on thermomechanical and mechanical properties such as *T*_g_ are poorly studied. In addition, the sensitivity of thermomechanical and mechanical measurement techniques to these heterogeneities has not been sufficiently investigated. Also, the study of the re-cross-linking of non-cross-linked areas and the possible relaxation of the network structure as part of the post-curing process at elevated temperatures is still poorly explored.

In this work, the influence of the process parameters of temperature and injection speed on the morphological properties such as IV, residual enthalpy, *T*_g_, and the storage modulus of two EMCs is shown. The effect of filler content on the detectability of the measurement methods is shown for two materials with an identical chemical composition but different SP contents of 10 wt%. The influence of the injection speed on the formation of a uniformly dense network is critically discussed. In addition, the effects on *T*_g_ of a post-mold-cure (PMC) process of 20 °C above the molding process temperature (+20 °C) are investigated.

## 2. Materials and Methods

### 2.1. Materials

**Figure 1:**
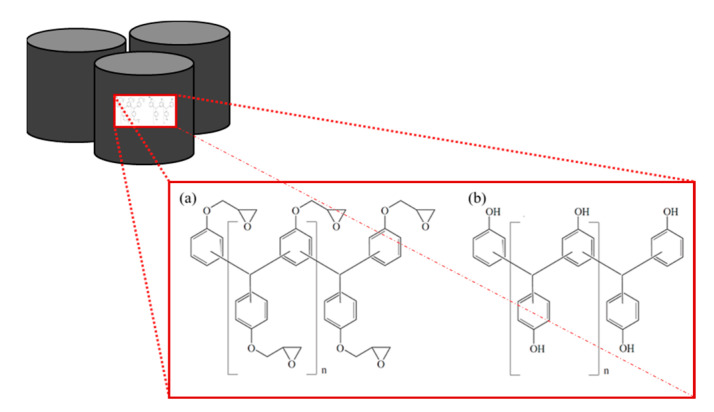
Multifunctional epoxy resin (**a**) and phenolic hardener (**b**) of the used pellets.

Two commercially premixed, latent-curing, epoxy resin molding compounds (EMC) containing a high filler loading of about 83 wt% (EMC 1) and 73 wt% (EMC 2) spherical silica particles (SP) with a nucleophilic reaction agent were used. The fundamental molecular structure of a multifunctional epoxy resin is described in Figure 1a and a multifunctional phenol hardener is displayed in Figure 1b. The EMCs have a *T*_g1_ of 220 °C at 100% curing state. Within the process window of 165 to 185 °C, the EMC is molded at a temperature below the final *T*_g1_. The material is pressed into pellets and was stored at 2 °C and warmed up to room temperature for >8 h prior to use.

The materials are used in the field of electronic packaging, where high *T*_g_s and a certain electrical insulation are required. 

### 2.2. Dielectric Analysis (DEA) in Transfermold

**Figure 2:**
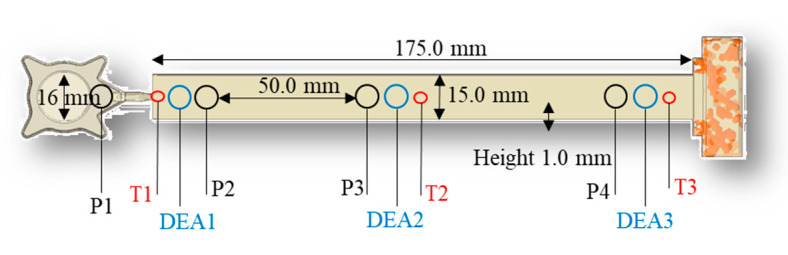
The geometry and location of the pressure sensors (P1–P4), dielectric analysis (DEA) sensors (DEA1–DEA3) and temperature sensors (T1–T3) are depicted.

The dielectric analysis (DEA) was performed with a 4/3RC monotrode (NETZSCH-Gerätebau GmbH, Selb, Germany) and a temperature sensor thermocouple type K (Kistler Instrumente AG, Winterthur, Switzerland), which were connected to a DEA analyzer (288 Epsilon, NETZSCH-Gerätebau GmbH, Selb, Germany). The sensors were implemented in a transfermold slit-mold cavity (175.0 × 15.0 × 1.0 mm). The location of the DEA and temperature sensors in the cavity are shown in Figure 2.

**Figure 3:**
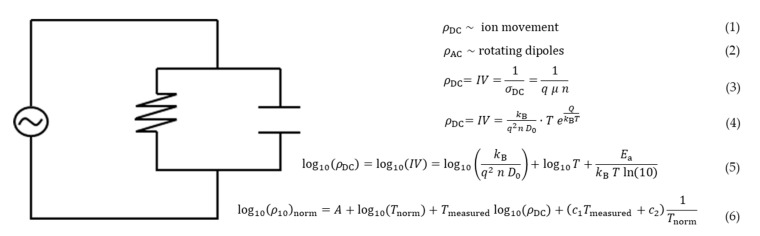
A regular circuit is shown with both an alternating current (AC) and a direct current (DC), and the derived equations are shown.

For polymers, the DEA is based on a measured conductivity caused by the contributors for an alternating current (AC) and direct current (DC), as shown in a regular circuit in Figure 3 [22].

In Equation (3) ${\;\sigma }_{DC}$ is the time-alternating conductivity (ohm^−1^·cm^−1^), *q* is the magnitude of electronic charge (coulombs), *μ*(*t*) is the free ion mobility (cm^2^/(V·s)), and *n* is the free ion concentration (cm^–3^) [22]. The value for the free ion movement is connected to the Stokes–Einstein equation. In Equation (4), $D_{0}$ is the diffusion coefficient (cm^2^/s), *k*_B_ the Boltzmann’s constant (eV/K), *T* is the absolute temperature (K), and Q is the heat quantity. By applying the natural logarithm, the DEA signal can be expressed as Equation (5). Two temperature coefficients are needed when comparing IV values of different temperatures, which are illustrated in Equation (6).

**Figure 4:**
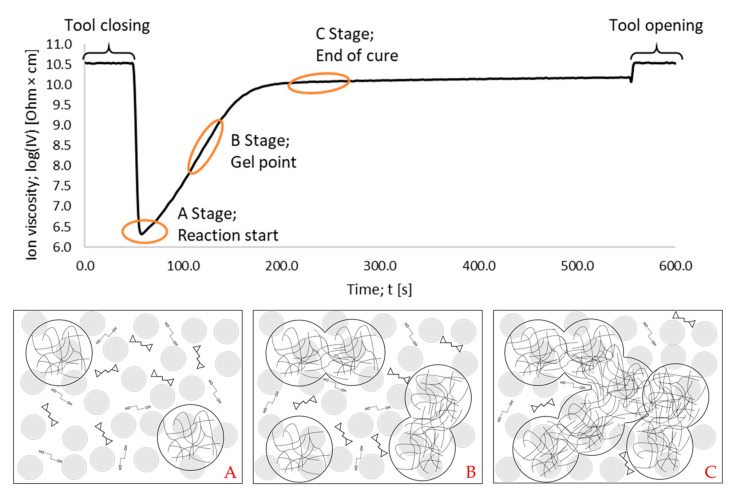
The DEA curve of a measurement at 175 °C is shown. Stage (**A**) represents the initiation steps at different discrete points and the monomers and oligomers in a free room containing silica particles (SP) (grey circles). Here, the first positive change in ion viscosity (IV) is observed. Stage (**B**) represents the creation of microgel clusters and gelation representing the highest reaction rate, and (**C**) illustrates the end of the cure, where the plateau of the DEA signal is reached.

A typical ion viscosity (IV) signal of the DEA, as well as the corresponding interpretation, are shown in Figure 4.

For further understanding, the DEA signal shown in Figure 4 must be interpreted at the molecular level and can be divided into three stages. Stage A describes the beginning of the reaction, where the reaction starts and initiation centers are built. Stage B represents the maximum reaction rate, which can be determined as the peak of the derivative. In Stage B, the movement of ions is more inhibited due to the multiple microgels that are formed and gelation starts. In Stage C, the material begins to form high-density polymer networks. This results in the maximum inhibition of ions during polymerization, causing the measured resistance to plateau and peak during the curing process. 

**Figure A1:**
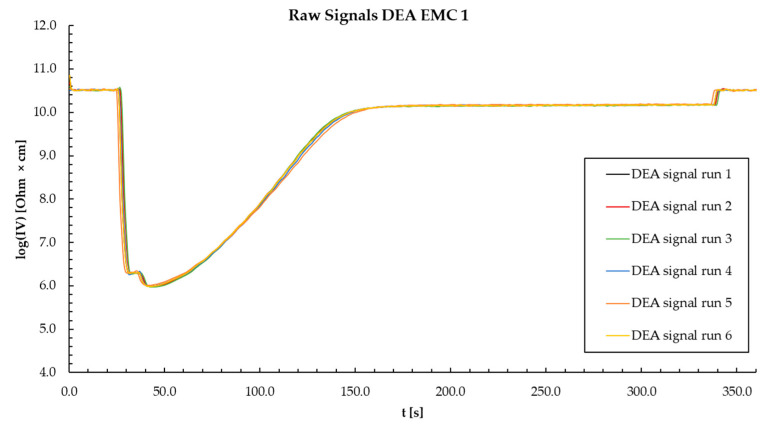
Six repetitions of the ion viscosity signal measured via DEA sensor 1 of the material EMC 1 at a temperature of 165 °C and an injection speed of 2.5 mm/s.

**Figure A2:**
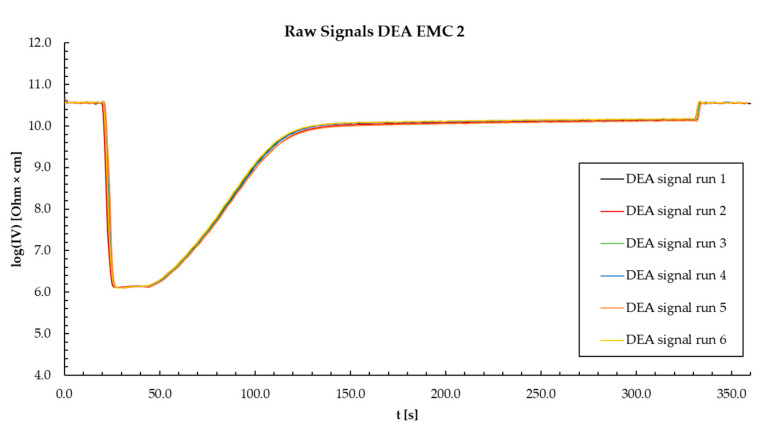
Six repetitions of the ion viscosity signal measured via DEA sensor 1 of the material EMC 2 at a temperature of 175 °C and an injection speed of 2.5 mm/s.

**Figure A3:**
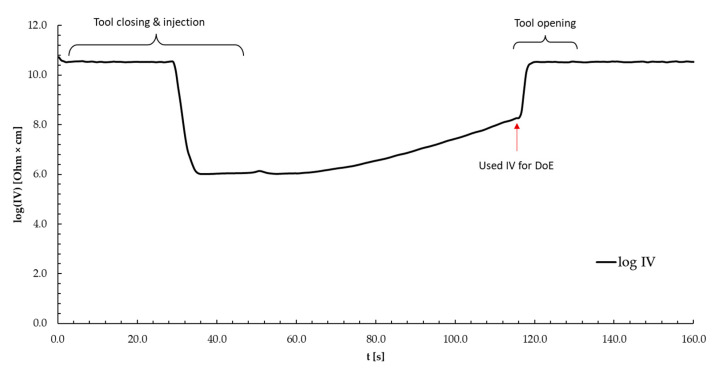
Determination of the maximum ion viscosity (red arrow) of the DEA signal used for the DoE analysis at a temperature of 165 °C, injection speed of 1 mm/s, and 60 s heating time.

The experiments were carried out with frequencies of 10 Hz and 100 Hz, which were found to create the lowest noise and ensure the highest reproducibility of the DEA signal. In addition, a change in frequency showed no effect on the conductivity. First, the reproducibility of the DEA signal was controlled by performing six isothermal measurements at different temperatures (165, 175, and 185 °C) and at different injection speeds (1.0, 2.5, and 4.0 mm/s), which were recorded for 5 min. Examples of the reproducibility of each material are provided in the Appendix A in Figure A1 and Figure A2. An example of detection of the IV for the design of experiment (DoE) is shown in Figure A3.

### 2.3. Design of Experiment (DoE)

**Figure 5:**
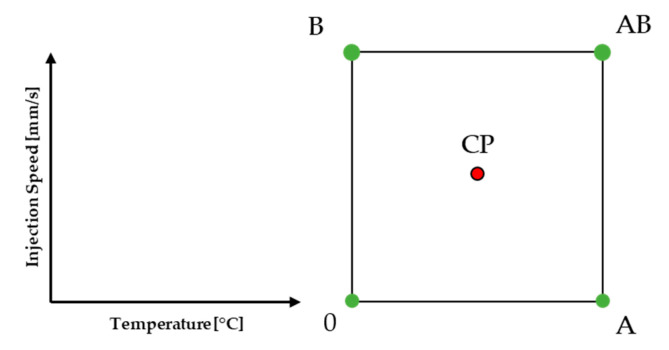
All experimental parameters are shown in a full factorial 2^2^ design of experiment (DoE) containing a center point (CP) and the varied transfermold process parameters temperature (A) and injection speed (B).

A design of experiment (DoE) was carried out to investigate the process window of the materials and to analyze the different effects of temperature and injection speed. Correlations with ion viscosity (IV), residual enthalpy, glass transition temperature (*T*_g_) at molding, *T*_g_ after a post-mold-cure (PMC) process, and storage modulus after PMC were taken as the responses. In addition, predictive models of all responses were calculated by a statistical evaluation of the design of experiment (DoE) data. The detailed factor level settings are listed in Section 3.2. The schematic space that was investigated is illustrated in Figure 5.

The center point (CP) presents the experiment that was conducted with general mean factor settings for all investigated factors, here 175 °C and 2.5 mm/s. The investigated temperatures were 165 °C, 175 °C, and 185 °C. The chosen injection speeds were 1.0 mm/s, 2.5 mm/s, and 4.0 mm/s. The limitations of the settings of the factors were determined on the basis of the processability of the material. The heating time for the tool of all experiments used for DoE was 60 s. The data obtained were analyzed utilizing Design Experts software (Stat-Ease, Inc., Minneapolis, MN, USA, Version 8.1.0).

### 2.4. Residual Enthalpy Measurements via Differential Scanning Calorimetry (DSC)

**Figure A4:**
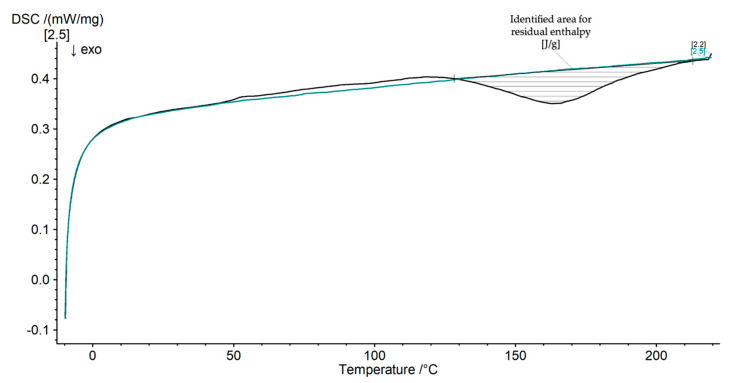
DSC measurement curves for the determination of the residual enthalpy, calculating the area between the first heating, shown as the data line (black line), and the second heating (green line).

Differential scanning colorimetry (DSC) measurements were carried out with a DSC 204F1 Phoenix^®^ (NETZSCH-Gerätebau GmbH, Selb, Germany) containing an integrated auto-sampler. All measurements were performed with a N_2_ flow rate of 40 mL/min, creating a nitrogen atmosphere. For each measurement, approximately 20.2 ± 0.6 mg was cut and weighed in aluminum crucibles (Concavus Pan and Lid from Al, NETZSCH-Gerätebau GmbH, Selb, Germany) from the molded samples near the DEA1 sensor. The used temperature profile was chosen from 20 to 260 °C, with a heating rate of 20 K/min. The rate of 20 K/min was chosen to improve the detection of the analytical signal when determining the residual enthalpy of highly filled composites. The differences in enthalpy were recorded and analyzed using the Proteus Thermal Analysis software (NETZSCH-Gerätebau GmbH, Selb, Germany, Version 7.1.0). An example of the detection of the residual enthalpy is shown in the Appendix A in Figure A4. In a last step, the obtained residual enthalpy was corrected to the total organic content of the EMC.

### 2.5. Determination of the Glass Transition Temperature (Tg) via Warpage Analysis

**Figure 6:**
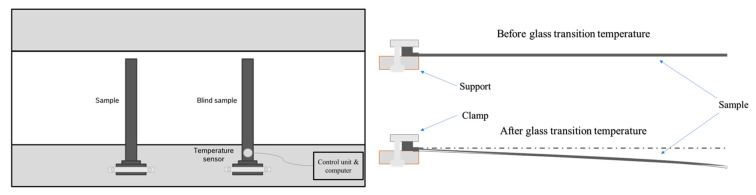
Schematic set-up of the determination of the *T_g_* directly after molding using a gravity-bending test during the heating of a sample within a warpage analyzer.

The principle of distortion analysis is based on the thermal expansion of the material. The expansions in the x, y, and z directions are measured at a given heating rate. The set-up and the scheme of the measurement are illustrated in Figure 6.

**Figure A5:**
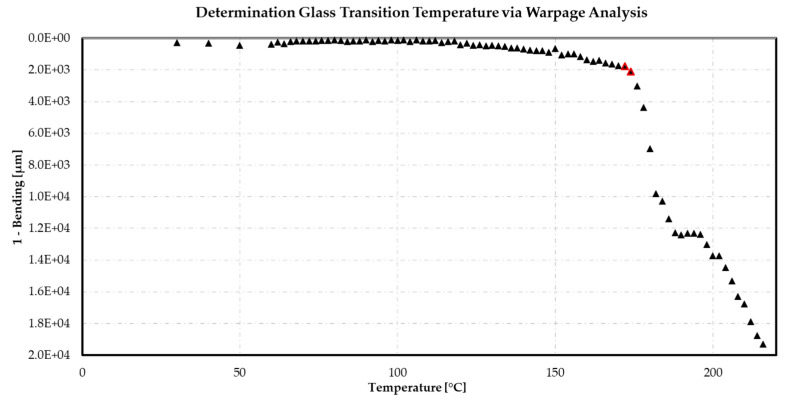
Glass transition temperature (*T*_g_), determined using a warpage analyzer. The onset of bending indicates that the *T*_g_ region was reached. *T*_g_ is defined as the temperature between two points (marked in red) at which the significant increase in bending starts.

The *T*_g_ was determined via the onset of bending created by gravity. One side of the samples was fixed in a heating chamber, comparable to a single-point bending analysis, and heated from 60 °C to 250 °C at a heating rate of 6 K/min. A reference sample containing a temperature sensor was clamped next to the investigated sample to control the temperature within the material without affecting the bending behavior. A diagram of the detection of the onset of the *T*_g_ is shown in Figure A5 in Appendix A.

### 2.6. Post-Mold-Cure (PMC)

The post-mold-cure (PMC) is used to post-cross-link the EMC material if this has not yet been achieved by the molding process. For this purpose, the investigated parts for the *T*_g_ measurements via DMA were stored + 20 °C above the process temperature for 4 h in a HeraTherm UT 6120/6 oven from Thermo Fisher Scientific (Thermo Fisher Scientific, Inc., Waltham, MA, USA) in order to reach the glass transition temperature (*T*_g_) and generate a post-cross-linking.

### 2.7. Dynamic Mechanical Analysis (DMA)

**Figure 7:**
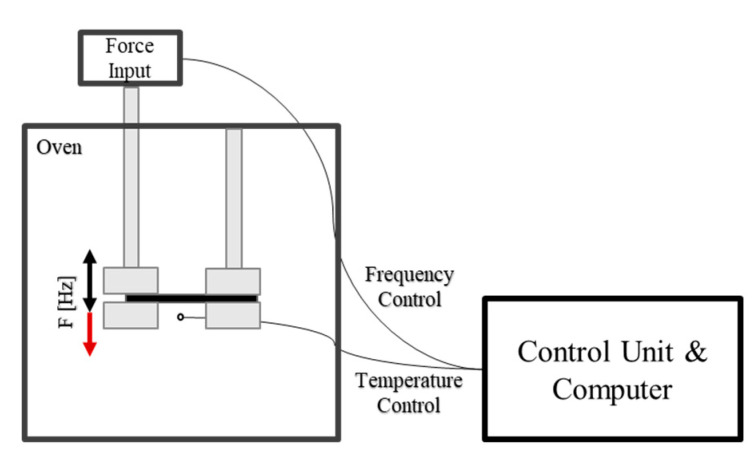
Sketch of the set-up for determining the *T_g_* via dynamic mechanical analysis (DMA) during heating in an oven at dynamic force input with a frequency of 1 Hz in a single-point bending method.

The DMA242 device from Netzsch (NETZSCH-Gerätebau GmbH, Selb, Germany) was used for all DMA experiments. The following program was used to determine the *T*_g_ and storage modulus. The sample was heated to 270 °C at the same rate of 2 °C/min. In a second step, the sample was cooled to 20 °C at the same rate and equilibrated at this temperature for 15 min. For each measurement, samples approximately 5.5 ± 0.5 mm wide were cut from the cured samples near the DEA1 sensor. The sample was then placed in the sample holder of the instrument, as shown in Figure 7, and measurements were taken.

**Figure A6:**
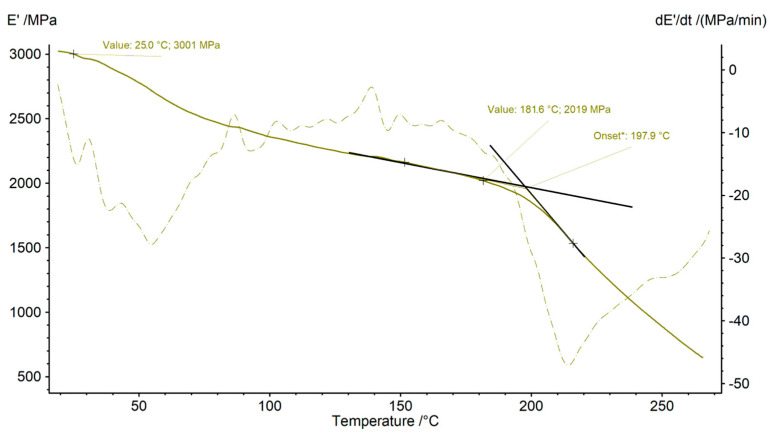
Example of the determination of the glass transition temperature (*T*_g_) and storage modulus via dynamic mechanical analysis (DMA) for the analysis of the design of experiment (DoE). *T_g_* determination was achieved using the first derivative of the storage modulus curve. Here, the onset, the start of *T_g_* region (181.6 °C) was used to illustrate the different effects of the parameter setting levels. The onset was used for comparison to the warpage measurement, where the onset was also used to calculate the *T_g_*. Storage modulus was determined at 25 °C; here 3002 MPa.

The measurement was analyzed with the Proteus 80 Thermal Analysis software (NETZSCH-Gerätebau GmbH, Selb, Germany, Version 8.0.3). The glass transition temperature (*T*_g_) was determined by placing two tangents in the region where the storage modulus changes rapidly and noting the onset of the *T*_g_ region through the dropdown from the upper tangent of the storage modulus. The storage modulus was determined at a temperature of 25 °C. A diagram of the determination of the onset of *T*_g_ and the storage modulus is given in the Appendix A in Figure A6.

## 3. Results

As mentioned above, before starting the dielectric analysis (DEA) experiments of the design of experiment (DoE), six repetitions were made each material at each temperature in order to prove the quality of DEA signal. Each DoE experiment was repeated three times and the center point was repeated five times. The process window was demonstrated in preliminary experiments: no demolding is possible below 160 °C and degradation by thermal decomposition occurs above 190 °C.

### 3.1. Effects of Injection Speed on Ion Viscosity (IV) Signal

**Figure A7:**
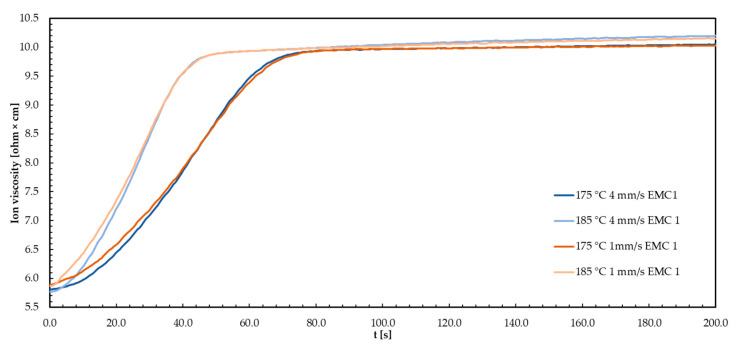
The ion viscosity (IV) curves for EMC 1 are shown during the transfer molding process with injection speeds of 1 mm/s (red curves) and 4 mm/s (blue curves) at 175 °C and 185 °C.

**Figure A8:**
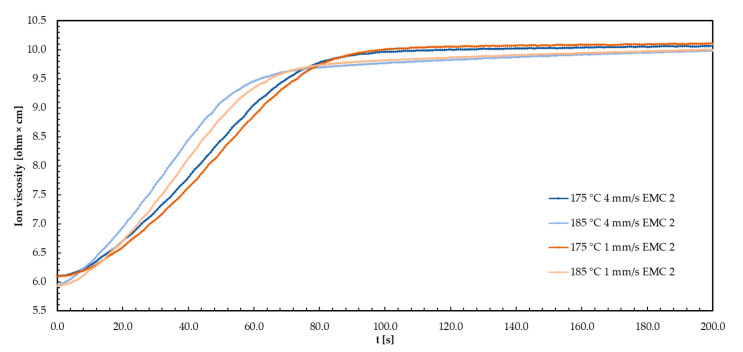
The ion viscosity (IV) curves for EMC 2 are shown during the transfer molding process with injection speeds of 1 mm/s (red curves) and 4 mm/s (blue curves) at 175 °C and 185 °C.

For a brief overview of the differences between the DEA signals regarding the injection speed, the measurements of each material at a temperature of 165 °C are shown. For all temperatures, a similar behavior is observed. The corresponding representative diagrams are listed in the Appendix A in Figure A7 and Figure A8.

**Figure 8:**
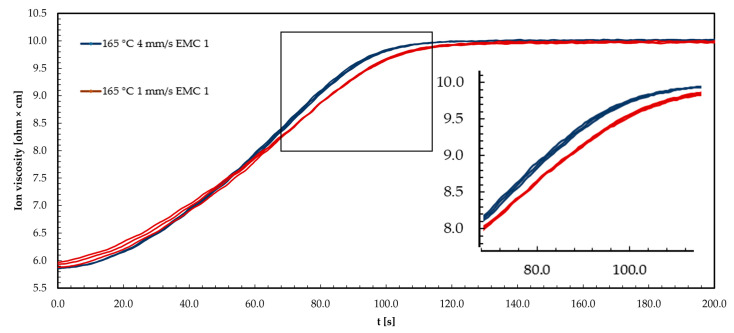
The ion viscosity (IV) curve of EMC 1 (73 wt% silica particle (SP)) is displayed during the transfer molding process at injection speeds of 1 mm/s (red curve) and 4 mm/s (blue curve) at 165 °C.

The graph in Figure 8 shows three example repetitions of DEA signals from EMC 1 at a process temperature of 165 °C. The ion viscosity (IV) curve is shown for injection speeds of 1 mm/s and 4 mm/s.

The ion viscosity (IV) curves for the two injection speeds of 1 mm/s and 4 mm/s show an increase as a function of time. The increase in IV can be explained by the increasing reaction process of the material at a temperature of 165 °C, which reduces the mobility of the ions. At an injection speed of 1 mm/s (red curve), the IV at the beginning of the reaction is higher than at 4 mm/s (blue curve). During the first 45 s of the measurement, the IV increases more rapidly. However, the maximum increase is lower, or the reaction rate is lower, compared to 4 mm/s. This shows the plateau of IV. As a result, the plateau of the IV signal is reached later at 1 mm/s than at 4 mm/s. Consequently, 4 mm/s shows a lower IV at the beginning of the reaction, but a slower increase to 45 s leads to a higher reaction speed and the plateau is reached more quickly. Reaching the plateau indicates the end of the reaction or, in the case of a material formed below the final *T*_g1_, the attainment of the glassy state during the process and thus the freezing of the ion mobility.

The shear heating of the molding compound explains the initially lower IV at 4 mm/s. This leads to a reduction in viscosity, and thus an increase in ion mobility. The energy input in the form of temperature results in a faster reaction process, and thus a higher maximum reaction rate and a faster achievement of *T*_g_ compared to 1 mm/s. The faster increase in IV at a 4 mm/s injection speed can be explained by an increase in the number of microgels or reaction centers, which favors ion confinement [35,36,39].

**Figure 9:**
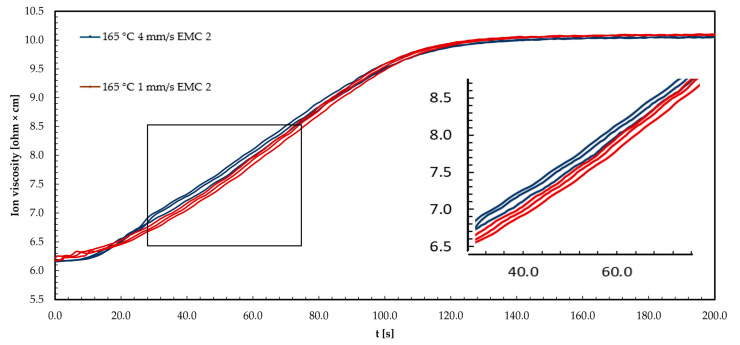
The ion viscosity (IV) for EMC 2 (83 wt% silica particle (SP)) is displayed during the transfer molding process at an injection speed of 1 mm/s (red curve) and 4 mm/s (blue curve) at 165 °C.

The following graph in Figure 9 shows three example repetitions of the DEA signals of EMC 2 at a process temperature of 165 °C. The IV curve is shown for injection speeds of 1 mm/s and 4 mm/s.

The IV curves shown for the two injection speeds of 1 mm/s and 4 mm/s show an increase with time. The increase in IV is due to the increasing reaction of the material at a temperature of 165 °C, which reduces the mobility of the ions. At an injection speed of 1 mm/s (red curve), the first 18 s of the measurement show a comparable IV to 4 mm/s (blue curve). After 18 s of measurement, a slower increase in IV is observed, which reaches a maximum gradient or reaction speed comparable to 4 mm/s over the course of the measurement. At an injection speed of 4 mm/s, the reaction slows down from about 60 s, as indicated by a reduced slope. The plateau of the IV signals at 1 mm/s and 4 mm/s is, therefore, reached simultaneously at the end of the reaction. Reaching the plateau indicates the end of the reaction or, in the case of a material deformed below the final *T*_g1_, the reaching of the glass state during the process and thus the freezing of the ion mobility. 

The faster initial increase in ion viscosity at 4 mm/s can be explained by the shear heating of the compound, comparable to EMC 1. The energy input in the form of temperature leads to a faster reaction process, and thus to a higher maximum reaction rate, which leads to the *T*_g_ being reached faster compared to 1 mm/s. A faster increase in IV at a 4 mm/s injection speed can be explained by an increase in the number of microgels or reaction centers, which favors the constriction of the ions [35,36,43]. The non-detectability of the decrease in IV can be explained by the higher proportion of silica particles (SP) in the molding compound (+10 wt%), which has a significant effect on viscosity and increased electrical insulation. This limits the detectability of these effects by the DEA sensors. The effect of electrical insulation is evident when considering the overall higher noise of the IV signals. The reduced increase in IV towards the end of the reaction at 4 mm/s supports the theory of interaction of reaction centers, which can produce a further steric hindrance effect due to the increased proportion of SP. In addition, the overall effect can be reduced to a minimum with this material due to the lower content of organic substances.

### 3.2. Results of the Design of Experiment (DoE)

The measured results of the investigated responses were analyzed based on the factor settings, including all repetitions, as shown in Table 1 and Table 2 for EMC 1 and EMC 2. An analysis of variances (ANOVA) was used to calculate significant effects and generate response surfaces. As previously described in Section 2.6, the post-mold-cure (PMC) process temperature was 20 °C higher than the mold process temperature.

#### 3.2.1. Response Surface of the Ion Viscosity (IV) of EMC 1 and EMC 2

An analysis of variances (ANOVA) was performed to determine the significant effects of the parameters, the interactions, and the response surface of the ion viscosity (IV), determined via DEA during the molding process of EMC 1. Based on the ANOVA results shown in Table 3, a response surface was calculated and is subsequently discussed.

An *F*-value of 592.08 implies that the model is significant and there is only a 0.01% chance that an *F*-value like this occurs due to noise. *P*-values of less than 0.0500 signify significant effects of the model terms A (temperature), B (injection speed), AB, and A^2^. A variation of less than 0.2 means that the predicted *R*^2^ of 0.9888 is in good agreement with the adjusted *R*^2^ of 0.9933. The adequate precision measures of the signal-to-noise ratio indicated an adequate signal with a value of 56.724, which is greater than 4.

In Table 4, the analysis of variances (ANOVA) is shown to determine the significant effects of the parameters, their interactions, and the response surface of the IV, determined via DEA during the molding process of EMC 2.

An *F*-value of 89,003 implies that the model is significant and there is only a 0.01% chance that an *F*-value like this occurs due to noise. *p*-values of less than 0.0500 signify significant effects of the model terms A (temperature), AB, and A^2^. However, model term B (injection speed) was not significant; this term is due to the hierarchical order. A variation of less than 0.2 means that the predicted *R*^2^ of 0.9926 is in good agreement with the adjusted *R*^2^ of 0.9955. The adequate precision measures of the signal-to-noise ratio indicated an adequate signal, with a value of 64.236, which is greater than 4.

**Figure 10:**
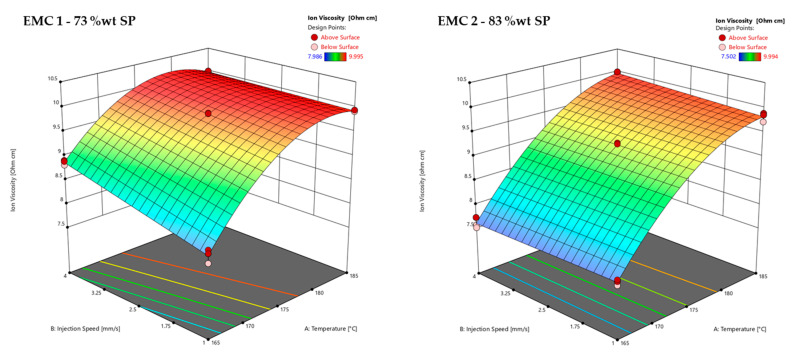
Response surface of the ion viscosity (IV), determined via DEA of EMC 1 (**left**) with 73 wt% silica particles (SP), and EMC 2 (**right**) with 83 wt% SP. Effects of transfer molding parameters A, temperature, and B, injection speed, on the IV (*z*-axis) are plotted. Dark red and light red dots depict the measured raw values above and below the response surface, respectively.

The effects of the temperature and injection speed on the ion viscosity (IV) of the DoE measured by DEA (*z*-axis) can be seen in Figure 10. An example of the determination of the maximum IV is shown in Appendix A in Figure A3.

Figure 10 shows the response surfaces of the ion viscosity (IV) values of EMC 1 (left) and EMC 2 (right) as a function of the process parameters of injection speed (B) and temperature (A) with a mold heating time of 60 s. EMC 1 shows a significant effect of injection speed, with an increase in IV between 165 °C and 175 °C. At about 180 °C, the plateau of IV is reached after a heating time of 60 s, meaning that the influence of the injection speed is no longer detected. The temperature shows a significant effect with a progressive increase in IV with temperature, which can be explained by the Arrhenius kinetic equation, in which temperature makes a significant contribution to the reaction progress. The degressive course of the IV is due to the significant factor A^2^, which calculates the quadratic course of the IV. EMC 2 shows no significant influence of the injection speed. The IV values are therefore the same after a heating time of 60 s, regardless of the injection speed. On the other hand, temperature shows a significant effect, which can also be explained by the Arrhenius kinetic equation. 

The non-detectability of the significant injection speed of EMC 2 in comparison to EMC 1 could be explained by the sensitivity of the DEA, which could be influenced by the higher silica particle (SP) content. A higher SP content results in an increase in electrical insulation. In addition, the thermal conductivity of the organic component is reduced by the increased SP. On the other hand, a lower organic content could lead to overall reduced effects. Consequently, thermal influences on the cross-linking reaction such as shearing can be minimized. Considering the observed effects of the injection speed on the total raw IV signal of Figure 9, it should be noted that these effects were not detected at significant levels due to the higher levels of noise. In addition, differences in the DEA signal during the curve could also indicate different kinetic behaviors, without effects on the final IV. Therefore, it should be assumed that the injection speed has an effect on the kinetics, which can lead to impacts on the thermo-mechanical and mechanical properties. 

#### 3.2.2. Response Surface of Residual Enthalpy of EMC 1 and EMC 2

On the basis of the analysis of variance (ANOVA) listed in Table 5, significant effects of the parameters, the interactions, and the response surface of the residual enthalpy determined via DSC after the molding process of EMC 1 are calculated and subsequently discussed.

An *F*-value of 396.97 represents a significant model. *p*-values of less than 0.0500 signify significant effects of the model terms A, B, AB, and A^2^. A variation of less than 0.2 means that the predicted *R*^2^ of 0.9832 is in good agreement with the adjusted *R*^2^ of 0.9900. The adequate precision measures of the signal-to-noise ratio indicated an adequate signal with a value of 47.927, which is greater than 4.

In Table 6, the results of the ANOVA are listed to calculate the significant effects of the parameters, their interactions, and the response surface of the residual enthalpy determined via DSC after the molding process of EMC 2.

An *F*-value of 562.64 represents a significant model. *p*-values of less than 0.0500 signify significant effects of the model terms A, B, and A^2^. A variation of less than 0.2 means that the predicted *R*^2^ of 0.9883 is in good agreement with the adjusted *R*^2^ of 0.9929. The adequate precision measurements of the signal-to-noise ratio indicated an adequate signal with a value of 53.209, which is greater than 4.

**Figure 11:**
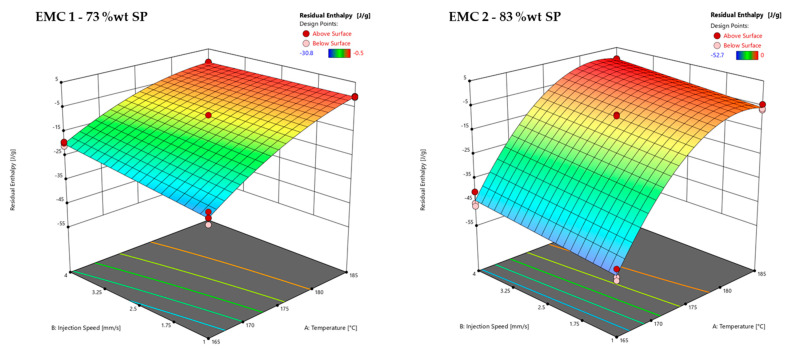
Response surface of the residual enthalpy measured via DSC of EMC 1 (**left**) with 73 wt% silica particles (SP) and EMC 2 (**right**) with 83 wt% SP. The effect of the transfer molding parameters A, temperature, and B, injection speed, on the residual enthalpy (*z*-axis) are plotted. Dark red and light red dots depict the measured raw values above and below the response surface, respectively.

The effects of the temperature and injection speed on the residual enthalpy determined by DSC (*z*-axis) are shown in Figure 11. A DSC measurement of a sample and the corresponding calculation of the residual enthalpy is illustrated in Appendix A in Figure A4. 

Figure 11 shows the residual enthalpy response surfaces of EMC 1 (left) and EMC 2 (right) as a function of the process parameters of injection speed (B) and temperature (A) with a mold heating time of 60 s. EMC 1 shows a significant influence of the injection speed, which is observed between 165 °C and 180 °C. At 183 °C, the material is fully cured after a heating time of 60 s, resulting in the effect of the injection speed no longer being detected. The significant effect of a progressive decrease in residual enthalpy is shown when increasing the temperature. This effect can also be explained by the Arrhenius kinetic equation, where temperature makes a significant contribution to the course of the reaction. EMC 2 shows the significant influence of the injection speed. This effect can be observed over the entire temperature range between 165 °C and 185 °C. Temperature shows a significant effect, with a progressive decrease in the residual enthalpy during an increase in temperature, which is also attributed to the Arrhenius kinetic equation.

The degressive course of the residual enthalpy of EMC 2 is due to the significant factor A^2^, which calculates the quadratic course of the residual enthalpy. The comparison of the two plots for EMC 1 and EMC 2 shows a significant influence of the injection speed on the cross-linking of both materials. In the case of EMC 2, this effect can be seen over the entire temperature scale of 165 °C and 185 °C, as the material is not fully reacted after 60 s. The amount of SP has a significant effect on the kinetics, which has been detailed in a previous study [13]. A higher proportion of SP results in greater thermal insulation and, thus, a slower reaction. The higher proportion of organic components in EMC 1 accelerates the reaction. The significant effects of injection speed support the assumption of thermal effects such as shearing on the cross-linking reaction. The lower effect of the injection speed in EMC 2 also indicates a minimizing effect due to the higher proportion of SP.

#### 3.2.3. Response Surface of Glass Transition Temperature (*T*_g_) after Molding of EMC 1 and EMC 2

On the basis of the analysis of variances (ANOVA) listed in Table 7, significant effects of the parameters, their interactions, and the response surface of the glass transition temperature (*T*_g_) after the molding process of EMC 1 are calculated and subsequently discussed.

An *F*-value of 324.86 represents a significant model. *p*-values of less than 0.0500 signify significant effects of the model terms A, B, AB, and A^2^. A variation of less than 0.2 means that the predicted *R*^2^ of 0.9827 is in good agreement with the adjusted *R*^2^ of 0.9878. The adequate precision measures of the signal-to-noise ratio indicated an adequate signal with a value of 43.180, which is greater than 4.

The ANOVA of EMC 2, listed in Table 8, is used to determine the significant effects of the parameters, their interactions, and the response surface of the *T*_g_ after the molding process.

An *F*-value of 66.17 represents a significant model. *p*-values of less than 0.0500 signify significant effects of the model terms A, B, and AB. A variation of less than 0.2 means that the predicted *R*^2^ of 0.9240 is in good agreement with the adjusted *R*^2^ of 0.9243. The adequate precision measures of the signal-to-noise ratio indicated an adequate signal with a value of 19.462, which is greater than 4.

**Figure 12:**
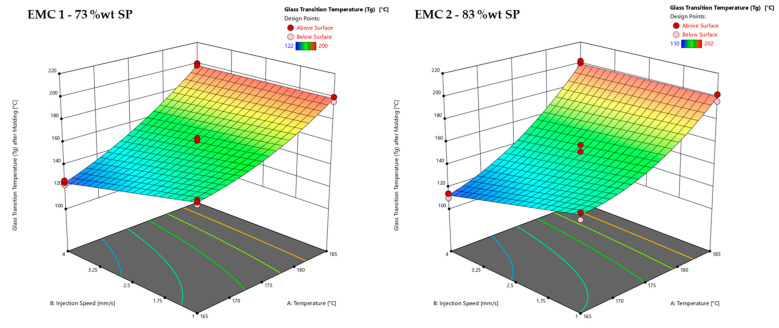
Response surface plot of the glass transition temperature (*T*_g_), determined via warpage analysis, is displayed for the materials EMC 1 (**left**), with 73 wt% silica particles (SP), and EMC 2 (**right**), with 83 wt% SP. Effects of transfer molding parameters A, temperature, and B, injection speed, on the *T*_g_ after molding (*z*-axis) are plotted. Dark red dots depict the measured raw values above the response surface; light red dots depict the measured raw values below the response surface.

The effects of the temperature and injection speed on the *T*_g_, determined by warpage analysis (*z*-axis), are shown in Figure 12. An example of the *T_g_* determination after molding is shown in Appendix A in Figure A5. 

Figure 12 shows the response surfaces of the glass transition temperatures (*T*_g_) after the molding process of EMC 1 (left) and EMC 2 (right) as a function of the process parameters of injection speed (B) and temperature (A) with a mold heating time of 60 s. EMC 1 and EMC 2 show a significant influence of injection speed and temperature. The two materials EMC 1 and EMC 2 show a very comparable *T*_g_ curve in the surface plots shown. A significant influence of the injection speed between 165 °C and 175 °C is observed. This is evident when looking at the measured *T*_g_ values after a process temperature of 165 °C, which are, on average, 12 °C higher for EMC 1 and 15 °C higher for EMC 2 when increasing the injection speed from 1 mm/s to 4 mm/s.

Overall, higher *T*_g_ values are obtained between 165 °C and 182 °C, which can be explained by the faster reaction of EMC 1 that was already observed. The process parameters of temperature and injection speed, therefore, have a significant influence on network formation. Furthermore, the *T*_g_ is not only dependent on the degree of cure, but also on the process temperature. The influence of the silica particles’ (SP) content on the measurement method can be seen via the comparatively greater increase in *T*_g_ values between 175 °C and 185 °C, which can be explained by the influence of material hardness. A higher proportion of SP results in a significantly higher hardness of the material, and therefore a later deflection of the sample. In addition, at approximately 182 °C and 183 °C, the warpage analysis method of measuring *T*_g_ shows its limitations in detecting very small differences, as the material has already completely reacted. The associated higher hardness of the material results in a delayed deflection of the sample during the measurement. 

The significant effects of injection speed confirm the influence of shearing on the cross-linking reaction and on network formation. The faster cross-linking due to shearing and the higher heating rates due to the injection speed generate multiple reaction centers, which lead to multiple microgel centers that can inhibit each other as the reaction progresses [35,36,37,38,39]. This can lead to an increase in heterogeneities, shown via lower *T*_g_ regions and an increased hardness [36,37,38]. These heterogeneities are irreversible and cannot be changed by further heating of the material, e.g., in a post-mold-curing (PMC) process; moreover, this effect could be extended [35]. 

#### 3.2.4. Response Surface of Glass Transition Temperature (*T*_g_) after PMC of EMC 1 and EMC 2

On the basis of the analysis of variance (ANOVA) listed in Table 9, the significant effects of the parameters, their interactions, and the response surface of the glass transition temperature (*T*_g_) after a post-mold-cure (PMC) process 20 °C above the molding process temperature of EMC 1 are calculated and subsequently discussed.

An *F*-value of 356.30 represents a significant model. *p*-values of less than 0.0500 signify significant effects of the model terms A, B, AB, and A^2^. A variation of less than 0.2 means that the predicted *R*^2^ of 0.9850 is in good agreement with the adjusted *R*^2^ *of* 0.9889. The adequate precision measures of the signal-to-noise ratio indicated an adequate signal with a value of 46.887 which is greater than 4.

The ANOVA of EMC 2 is listed in Table 10 and used to determine the significant effects of the parameters, the interactions, and the response surface of the *T*_g_ after a post-mold-cure (PMC) process 20 °C above the molding process temperature.

An *F*-value of 49.18 represents a significant model. *P*-values of less than 0.0500 signify significant effects of the model terms A and A^2^. However, model term B (injection speed) is not significant; this term is calculated in the model to illustrate the differences between EMC 1 and EMC 2. The predicted *R*^2^ of 0.8577 shows low agreement since the difference from the adjusted *R*^2^ of 0.900 is bigger than 0.2. The predictive accuracy of this model must therefore be viewed critically, as the predicted values can deviate considerably from the actual measured values. However, the surface representation with a high adjusted R^2^ of 0.9003 is suitable for illustrating the results. The adequate precision measures for the signal-to-noise ratio indicated an adequate signal with a value of 14.516, which is greater than 4.

**Figure 13:**
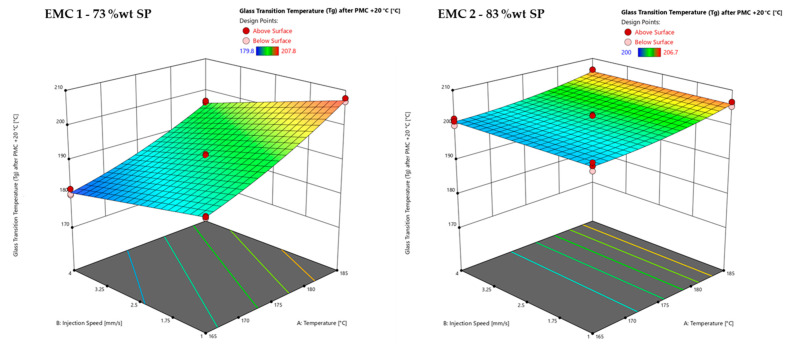
Response surface of the glass transition temperature (*T*_g_), determined via dynamic mechanical analysis (DMA) after a post-mold-cure (PMC) process 20 °C above the molding process temperature of EMC 1 (**left**), with 73 wt% silica particles (SP), and EMC 2 (**right**), with 83 wt% SP, is shown. Effects of transfer molding parameters A, temperature, and B, injection speed, on the *T*_g_ after molding (*z*-axis) are plotted. Dark red dots depict the measured raw values above the response surface; light red dots depict the measured raw values below the response surface.

The effects of the temperature and injection speed on the *T*_g_ after a post-mold-cure (PMC) process 20 °C above the molding temperature (*z*-axis), determined via DMA, are shown in Figure 13. An example of the DMA measurement of a test point and the determination of the *T*_g_ is shown in Appendix A, Figure A6**.**

Figure 13 shows the response surfaces of the *T*_g_ after a post-mold-cure (PMC) process 20 °C above the cavity pressure temperature of EMC 1 (left) and EMC 2 (right), as a function of the process parameters of injection speed (B) and temperature (A), with a heating time in the mold of 60 s. EMC 1 shows the significant influence of injection speed and temperature. The significant influence of injection speed can be seen through the tilting of the surface plot. Increasing the temperature from 165 °C to 185 °C increases the effect of injection speed from a difference of 8 °C to a difference of 15 °C. The still significant effect of injection speed after the PMC process illustrates the irreversibility of heterogeneous network formation. The increase in temperature shows the dependence of *T*_g_ on the process temperature. At 165 °C and a PMC temperature of 185 °C, a *T*_g_ of 188 °C is obtained at an injection speed of 1 mm/s. At 185 °C and a PMC temperature of 205 °C, the *T*_g_ is 207 °C. 

EMC 2 shows a significant temperature effect. Increasing the temperature of the PMC process leads to an increase in *T*_g_. Injection speed has no significant effect. This is apparently because the increased silica particle (SP) content in the material has a significant effect on the DMA measurement signal. The significantly higher hardness due to the higher SP content leads to a delayed softening of the sample, and thus to a shift in *T*_g_, similar to the warpage determination of *T*_g_ at highly cross-linked samples. In addition, the thermal insulation provided by the SP may have resulted in a delayed *T*_g_ determination. It should also be noted that, at lower PMC temperatures, the shift in *T*_g_ increases due to post-reactions during the DMA measurement. In addition, the overall effect can be reduced to a minimum when there are higher amounts of SP in the material due to the lower content of organic substances. 

**Figure 14:**
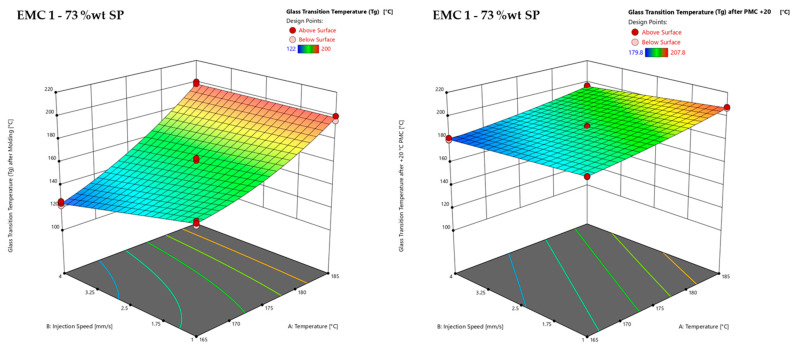
Response surface of the glass transition temperature (*T*_g_), determined via warpage analysis, is shown for the materials EMC 1 (**left**), with 73 wt% silica particles (SP), and the response surface of the glass transition temperature (*T*_g_) determined via dynamic mechanical analysis (DMA) after a post-mold-cure (PMC) process 20 °C above the molding process temperature of EMC 1 (**right**) with 73 wt% silica particles (SP). Effects of transfer molding parameters A, temperature, and B, injection speed, on the *T*_g_ after molding (*z*-axis). Dark red dots depict the measured raw values above the response surface; light red dots depict the measured raw values below the response surface.

In Figure 14, the effect of PMC is illustrated, showing a comparison of the *T*_g_ values directly after molding (left) and after a PMC process 20 °C above the molding temperature (right).

Comparing the *T*_g_ values after molding (left) with the *T*_g_ values after a PMC process 20 °C above the molding temperature, a subsequent temperature increase of 20 °C in a PMC process increases the *T*_g_ value, indicating a post-mold reaction, although the samples of a process temperature of 185 °C had already completely reacted according to the DEA and DSC signals. As already discussed, DEA shows the end of the reaction, after a plateau in the ion viscosity is reached, which represents the achievement of the glassy state during the molding process. In DSC analysis, the residual enthalpy is detected, which illustrates possible residual or post reactions. However, during the heating phase of the DSC measurements, the material is already reacting, suppressing exothermic signals, which results in non-detectability. This is especially valid for samples at highly cross-linked states or inorganic highly filled materials. For this reason, no residual enthalpy was detected in most samples at a process temperature of 185 °C. 

#### 3.2.5. Response Surface of Storage Modulus after PMC of EMC 1 and EMC 2

On the basis of the results of the analysis of variance (ANOVA) listed in Table 11, the significant effects of the parameters, their interactions, and the response surface of the storage modulus after a post-mold-cure (PMC) process 20 °C above the molding process temperature of EMC 1 are calculated and subsequently discussed.

An *F*-value of 47.80 represents a significant model. *p*-values of less than 0.0500 signify significant effects of the model terms B and AB. However, model terms A (temperature) are not significant; this term is due to the hierarchical order. The predicted *R*^2^ of 0.8432 shows low agreement since the difference between the adjusted *R*^2^ of 0.8977 is bigger than 0.2. The predictive accuracy of this model must therefore be viewed critically as the predicted values can deviate considerably from the actual measured values. However, the surface representation with a high adjusted *R*^2^ of 0.8977 is suitable for illustrating the results. The adequate precision measures for the signal-to-noise ratio indicated an adequate signal with a value of 17.404, which is greater than 4.

The ANOVA listed in Table 12 is used to calculate the significant effects of the parameters, the interactions, and the response surface of the storage modulus after a post-mold-cure (PMC) process 20 °C above the molding process temperature of EMC 2.

An *F*-value of 10.74 represents a significant model. *p*-values of less than 0.0500 signify significant effects of the model terms A and B. The predicted *R*^2^ of 0.4263 shows low agreement since the difference between the adjusted *R*^2^ of 0.5490 is bigger than 0.2. The predictive accuracy of this model is bad and the surface representation, with a high adjusted R^2^ of 0.5490, is not suitable for illustrating the results. Therefore, only the response surface of EMC 1 is subsequently shown and consequently discussed. The adequate precision measure of the signal-to-noise ratio, at 8.995, is greater than 4, indicating an adequate signal.

**Figure 15:**
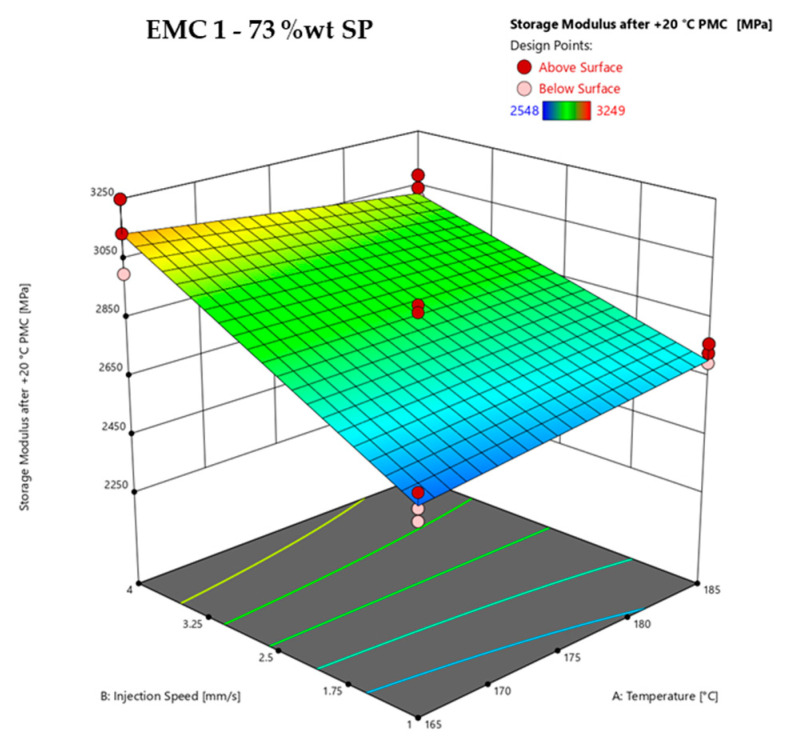
Response surface of the storage modulus after a post-mold-cure (PMC) process 20 °C above the molding process temperature of EMC 1 (73 wt% silica particles (SP)), calculated via DMA, is shown. Effect of transfer molding parameters A, temperature, and B, injection speed, on the storage modulus (*z*-axis) are plotted. Dark red dots depict the measured raw values above the response surface; light red dots show the measured raw values below the response surface.

The effects of the temperature and injection speed on the storage modulus after a post-mold-cure (PMC) process (*z*-axis), determined via DMA, are shown in Figure 15. An example of the determination of the storage modulus at 25 °C is shown in Appendix A in Figure A6. 

Figure 15 shows the response surface of the storage modulus after a post-mold-cure (PMC) process 20 °C above the mold temperature of EMC 1 as a function of the process parameters of injection speed (B) and temperature (A) with a mold heating time of 60 s. EMC 1 shows the significant influence of injection speed. This effect is observed in the slope of the surface plot over the entire investigated process window. Increasing the injection speed from 1 mm/s to 4 mm/s increases the storage modulus by 600 MPa at a process temperature of 165 °C and by 250 MPa at a process temperature of 185 °C. The significant interaction that was calculated between temperature and injection speed shows a slight decrease in storage modulus at 4 mm/s and a slight increase at 1 mm/s as the temperature increases from 165 °C to 185 °C.

EMC 2 shows a bad predictive accuracy of the model, with very high standard deviations of the measurement results. The high deviations within a test point are explained by the significantly higher proportion of silica particles (SP). Considering Table 1 and Table 2, the highest measured storage modulus value of 4545 MPa of EMC 2 represents a material that is 1296 MPa harder than EMC 1, at 3249 MPa. With 83 wt% SPs in the material, it could be assumed that the SPs are not homogeneously distributed throughout the material and can significantly affect the mechanical DMA, especially when measuring very small samples. A larger storage modulus could provide more information about the effects or heterogeneities. Differentiated gate designs could also be used to investigate different SP distributions. In addition, the heterogeneity of the polymer network may increase the uneven distribution of SP due to the formation of microgel clusters.

Irrespective of the effect of the silica particles (SP), the hypothesis that an inhomogeneous or heterogeneous polymer network is generated by increasing the injection speed can be assumed based on the diagram shown in Figure 15. The generation of multiple microgel clusters by increasing the injection speed, which, as previously mentioned, is accompanied by the faster heating of the material and additional shear heating, leads to steric hindrance during the reaction, and thus to multiple cross-linking clusters. This heterogeneous polymeric network leads to higher stress in the material, resulting in a reduced *T*_g_ and an increased storage modulus, as discussed in Section 3.2.3 and Section 3.2.4. As this effect is still visible after PMC, it can be considered that the heterogeneity is irreversible. 

## 4. Discussion

The curing of an epoxy–phenol resin is temperature-controlled and characterized by two independent phenomena: gelation and vitrification [34]. The significant effect of temperature and injection speed on the ion viscosity (IV) and residual enthalpy illustrated the temperature-controlled curing of epoxy phenolic resins. Increasing the injection speed led to a higher heating rate and higher shear warming, as well as a shortened gel time during curing. 

The reaction of a chain polymerization begins with initiation steps that begin at different discrete points in the system, which is also illustrated in Figure 4 [35]. This process is inherently inhomogeneous or heterogeneous and can develop nanoscale heterogeneities known as microgels [36]. As polymerization progresses, multiple microgels or reaction centers can take the form of cross-linked polymer spirals swollen by unreacted monomers and oligomers. The dimensions and concentration of the polymer coils increase until a cross-linked structure is formed. This penetrates the system and leads to macrogelation [48,49,50,51]. Controlling the temperature through the process parameters accelerates the reaction and leads to increased microgel formation due to the reduced gel time. Multiple microgels or reaction centers essentially affect the gelation and thus the macroscopic behavior of the system, and consequently lead to inhomogeneous or heterogenous polymerization. 

**Figure 16:**
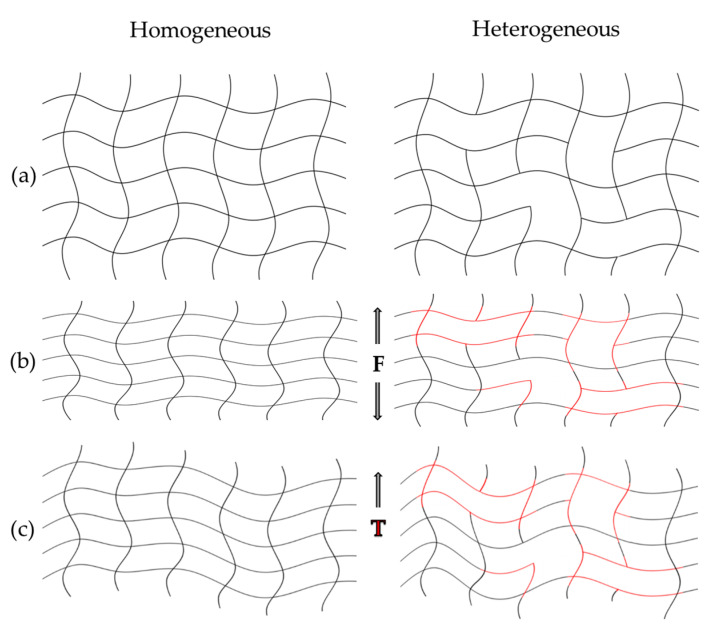
Schematic representation of a homogeneous and heterogeneous polymer network (**a**) and their different effects in relation to external influences such as force (**b**) and temperature (**c**).

By evaluating the design of experiments (DoE), the influence of temperature and injection speed on morphological properties was demonstrated, resulting in different *T_g_* and storage moduli. These results are in good agreement with the observations of the literature, where thermal effects such as shearing exert a significant influence on the final morphological properties of the polymer network [35,36,37,38,39,40,41,42,43,44,45]. Compared to a homogeneous network, a heterogeneous network has less densely cross-linked areas. The heterogeneous structure can therefore react differently to external influences such as force and temperature, as shown schematically in Figure 16.

Homogeneous polymer networks are more resistant, as forces can be distributed evenly across the structure. Heterogeneous networks, on the other hand, are stiffer and more brittle, as the influencing forces can only be distributed unevenly, resulting in a higher storage modulus, for example. Homogeneous networks can also distribute thermal energy more evenly, which leads to a higher *T_g_*. Heterogeneous systems have a higher chain mobility in their less dense areas, which leads to a lower *T_g_*. The total *T_g_* range of heterogeneous systems is therefore larger and the onset is lower [35,36,38,39,41,42,43,45].

Consequently, the observed decrease in *T_g_* measurements determined by warpage analysis (Figure 12) and DMA (Figure 13), as well as the increased storage modulus (Figure 15) indicates the formation of a heterogeneous polymer network due to an accelerated cross-linking reaction caused by increased microgel cluster formation generated by the faster heating and shear heat effects. 

In contrast to gelation, vitrification is not independent of the temperature, since the thermoset polymer fragments’ ability to generate cooperative motion increases with the temperature. Vitrification is independent of the degree of cure and is always a reversible process, whereas gelation, for example, in epoxy–phenolic resins, cannot be reversed. Another significant difference to the gel point is that the glass transition significantly inhibits the curing process [52,53]. In the glassy state, the free space is significantly reduced, and the mobility of chains and molecules is inhibited, which also reduces the mobility of functional reactive groups and quickly stops the reaction. [35] However, polymerization can be resumed by increasing the temperature as long as the conversion is not complete [35,38,54]. 

The significant increase in *T_g_* after the post-mold-cure (PMC) experiments illustrated the resumed polymerization of the materials, which can be analyzed through comparing the *T_g_* values measured by warpage analysis and DMA (Figure 14). Furthermore, the results of the *T_g_* evaluation using DMA showed that the set temperature in the process also represents the *T_g_* as long as the *T_g_* is not equal to *T_g_*_1_ (100% reacted). 

The influence of silica particles (SPs) on the microstructural quality of the thermosets and their effects on the sensitivity of material characterization methods have been poorly studied, and heterogeneous effects have only been suspected [7,14]. 

Therefore, for the materials analyzed in this work, it must be noted that silica particles (SP) with a proportion of 73 wt% to 83 wt% are additionally embedded in such structures, as shown in Figure 16. It can be assumed that the SPs are also heterogeneously distributed in the system, and therefore cause fluctuations in the measurement results, as observed for the evaluation of the storage modulus for the 83 wt% filled material. The effects on electrical insulation and thermal conductivity, as well as the reduction in organic content with increasing SPs, could therefore lead to a lower sensitivity of DEA (Figure 10) and DSC (Figure 11). In addition, highly filled materials with a high degree of cure tend to be extremely stiff. As a consequence, it is more difficult to verify the *T_g_* as this requires more energy for chain mobility, shifting the *T_g_* to higher values. The significant increase in the *T_g_* gradient at highly cross-linked states, and the significantly higher storage modulus due to 10 wt% SP, emphasized this effect. 

## 5. Conclusions

In the present work, the in-line monitoring of the curing behavior via dielectric analysis (DEA) and the corresponding morphological characteristics of residual enthalpy, glass transition temperature (*T*_g_), and the storage modulus of two epoxy molding compounds (EMCs) are evaluated. The ion viscosity (IV) signals of EMC1 and EMC2 showed significant differences by increasing the injection speed from 1 mm/s to 4 mm/s. The design of experiment (DoE) approach showed that injection speed has a significant effect on IV, residual enthalpy, *T*_g_ after molding and after post-mold-cure (PMC), and storage modulus after PMC. Increasing the injection speed from 1 mm/s to 4 mm/s resulted in a significant increase in IV and decrease in residual enthalpy, a decrease in *T*_g_ up to 15 °C, and an increase in storage modulus of 600 MPa for EMC 1. It was shown that the reaction of EMC 1 and EMC 2 restarts in a PMC process, even if stagnation of the reaction is detected with the IV reaching a plateau and no residual enthalpy. In PMC, the reaction process starts when the temperature reaches *T*_g_ (*T*_g_ = *T*_process_), leading to an increase in *T*_g_ until the final *T*_g_ (*T*_g1_) is reached. Furthermore, by using the warpage measurement method for *T*_g_ detection, it has been shown that *T*_g_ = *T*_process_ if the material has not yet reached the final *T*_g_. The sensitivity limits of the DEA and DSC measurements were illustrated by varying the 10 wt% silica particles (SP) content of the analyzed materials. As a result, the higher SP content in the material, 83 wt% compared to 73 wt%, showed that there was no significant effect of injection speed on the cross-linking states determined by DEA, although effects on residual enthalpy and *T*_g_ were observed for both materials. Therefore, DEA is a useful tool to identify heterogeneities as a function of process parameters, as long as the effects are large enough. A comparison of the *T*_g_ measurement methods used showed the limited detectability of the DMA and warpage analysis methods in highly filled materials in high cure states. The observations of all measurement methods led to the assumption that increasing the injection speed leads to an accelerated cross-linking reaction due to increased microgel cluster formation, resulting in a lower *T*_g_ and an increased storage modulus. The effects of the increased, uneven distribution of SP due to the high injection speed need to be investigated by varying the gate geometry of the molding tool. The effects need to be verified on larger samples to investigate the macroscale effect. The application of a warpage analysis for *T*_g_ determination needs to be translated to larger samples to demonstrate a direct use case for molded products.

## Figures and Tables

**Table 1:** Factor-level settings and the investigated responses values of the design of experiment (DoE) evaluation of EMC 1.

	Factor 1	Factor 2	Response 1	Response 2	Response 3	Response 4	Response 5
Run	A: Temperature [°C]	B: Injection Speed [mm/s]	Ion Viscosity [Ω·cm]	Residual Enthalpy [J/g]	Glass Transition Temperature (*T_g_*) after Molding [°C]	Glass Transition Temperature (*T_g_*) after PMC [°C]	Storage Modulus after PMC [MPa]
1	165	1	8.243	−25.978	154	188.0	2638
2	165	1	8.174	−28.252	150	188.6	2548
3	165	1	7.986	−30.800	152	188.3	2588
4	185	1	9.919	−0.001	200	207.8	2735
5	185	1	9.939	−0.001	200	206.9	2767
6	185	1	9.911	−0.001	196	206.8	2701
7	165	4	8.913	−19.563	122	181.6	3002
8	165	4	8.891	−20.796	124	179.8	3136
9	165	4	8.817	−19.074	126	180.1	3249
10	185	4	9.995	−0.001	200	195.0	3006
11	185	4	9.973	−0.001	196	196.2	3089
12	185	4	9.966	−0.001	198	196.7	3042
13	175	2.5	9.882	−8.100	162	191.8	2899
14	175	2.5	9.866	−8.593	154	191.9	2786
15	175	2.5	9.834	−8.459	164	191.5	2845
16	175	2.5	9.871	−8.448	160	191.3	2807
17	175	2.5	9.838	−8.537	164	191.0	2873

**Table 2:** Factor-level settings and the investigated responses values of the design of experiment (DoE) evaluation of EMC 2.

	Factor 1	Factor 2	Response 1	Response 2	Response 3	Response 4	Response 5
Run	A: Temperature [°C]	B: Injection Speed [mm/s]	Ion Viscosity [Ω·cm]	Residual Enthalpy [J/g]	Glass Transition Temperature (*T_g_*) after Molding [°C]	Glass Transition Temperature (*T_g_*) after PMC [°C]	Storage Modulus after PMC [MPa]
1	165	1	7.647	−48.118	144	201.4	3015
2	165	1	7.683	−51.294	138	200.1	3236
3	165	1	7.572	−52.706	144	202.3	2866
4	185	1	9.716	−0.004	202	206.0	4545
5	185	1	9.889	−6.588	202	205.4	4357
6	185	1	9.855	−6.141	196	206.7	4049
7	165	4	7.560	−45.371	114	202.0	2537
8	165	4	7.502	−46.647	114	201.2	2508
9	165	4	7.724	−40.535	110	200.0	2543
10	185	4	9.969	0.000	196	206.5	4002
11	185	4	9.928	0.000	200	205.5	2368
12	185	4	9.994	0.000	202	205.9	3024
13	175	2.5	9.289	−9.541	150	202.9	3740
14	175	2.5	9.265	−10.647	146	202.1	3686
15	175	2.5	9.211	−8.541	158	203.0	2456
16	175	2.5	9.241	−9.059	148	202.8	2874
17	175	2.5	9.234	−9.488	152	202.3	3452

**Table 3:** Analysis of variances (ANOVA) of the DoE response surface models of the response ion viscosity (IV) of EMC 1, including degrees of freedom (d*f*), the ratio of two mean squares (*F*-value), and the null hypothesis significance test of 0.05 (*p*-value).

Source	Sum of Squares	d*f*	Mean Square	*F*-Value	*p*-Value
Model	8.5100	4	2.1300	592.08	<0.0001
A—Temperature	6.2800	1	6.2800	1747.59	<0.0001
B—Injection Speed	0.4732	1	0.4732	131.75	<0.0001
AB	0.3512	1	0.3512	97.79	<0.0001
A^2^	1.4100	1	1.4100	391.18	<0.0001
Pure Error	0.0431	12	0.0036		

**Table 4:** Analysis of variance (ANOVA) of the evaluated DoE response surface models of the response IV of EMC 2, including degrees of freedom (d*f*), ration of two mean squares (*F*-value), and the null hypothesis significance test of 0.05 (*p*-value).

Source	Sum of Squares	d*f*	Mean Square	*F*-Value	*p*-Value
Model	16.4500	4	4.1100	890.03	<0.0001
A—Temperature	15.5500	1	15.5500	3365.81	<0.0001
B—Injection Speed	0.0082	1	0.0082	1.77	0.2078
AB	0.0250	1	0.0250	5.40	0.0385
A^2^	0.8647	1	0.8647	187.12	<0.0001
Pure Error	0.0555	12	0.0046		

**Table 5:** Analysis of variance (ANOVA) of the obtained DoE response surface models of the response residual enthalpy calculated via DSC, including degrees of freedom (d*f*), ration of two mean squares (F-value) and the null hypothesis significance test of 0.05 (*p*-value).

Source	Sum of Squares	d*f*	Mean Square	*F*-Value	*p*-Value
Model	1785.75	4	446.44	396.97	<0.0001
A—Temperature	1619.09	1	1619.09	1439.70	<0.0001
B—Injection Speed	52.90	1	52.90	47.04	<0.0001
AB	56.32	1	56.32	50.08	<0.0001
A^2^	57.44	1	57.44	51.07	<0.0001
Pure Error	13.50	12	1.12		

**Table 6:** Analysis of variance (ANOVA) of the DoE response surface models of the response residual enthalpy of EMC 2, including degrees of freedom (d*f*), ration of two mean squares (*F*-value), and the null hypothesis significance test of 0.05 (*p*-value).

Source	Sum of Squares	d*f*	Mean Square	*F*-Value	*p*-Value
Model	6948.83	4	1737.21	562.64	<0.0001
A—Temperature	5966.96	1	5966.96	1932.54	<0.0001
B—Injection Speed	111.92	1	111.92	36.25	<0.0001
A^2^	869.42	1	869.42	281.58	<0.0001
Pure Error	37.05	12	3.09		

**Table 7:** Analysis of variances (ANOVA) of the obtained DoE response surface models of the response glass transition temperature (*T*_g_), determined via warpage measurements, including degrees of freedom (d*f*), ration of two mean squares (*F*-value), and the null hypothesis significance test of 0.05 (*p*-value).

Source	Sum of Squares	D*f*	Mean Square	*F*-Value	*p*-Value
Model	17,556.93	4	4389.23	324.86	<0.0001
A—Temperature	15,696.33	1	15,696.33	1161.74	<0.0001
B—Injection Speed	675.00	1	675.00	49.96	<0.0001
AB	616.33	1	616.33	45.62	<0.0001
A^2^	569.26	1	569.26	42.13	<0.0001
Pure Error	162.13	12	13.51		

**Table 8:** Analysis of variances (ANOVA) of the obtained DoE response surface models of the response glass transition temperature (*T*_g_), measured via a warpage analysis of EMC 2 including degrees of freedom (d*f*), ration of two mean squares (*F*-value), and the null hypothesis significance test of 0.05 (*p*-value).

Source	Sum of Squares	d*f*	Mean Square	*F*-Value	*p*-Value
Model	16,987.67	3	5662.56	66.17	<0.0001
A—Temperature	15,696.33	1	15,696.33	183.41	<0.0001
B—Injection Speed	675.00	1	675.00	7.89	0.0148
AB	616.33	1	616.33	7.20	0.0188
Pure Error	954.13	12	79.51		

**Table 9:** Analysis of variance of the obtained DoE response surface models of the response glass transition temperature (*T*_g_), determined via a dynamic mechanical analysis (DMA) of EMC 1, including degrees of freedom (d*f*), ration of two mean squares (*F*-value), and the null hypothesis significance test of 0.05 (*p*-value).

Source	Sum of Squares	d*f*	Mean Square	*F*-Value	*p*-Value
Model	12,288.53	4	3072.13	356.30	<0.0001
A—Temperature	10,920.33	1	10,920.33	1266.53	<0.0001
B—Injection Speed	616.33	1	616.33	71.48	<0.0001
AB	560.33	1	560.33	64.99	<0.0001
A^2^	191.53	1	191.53	22.21	0.0005
Pure Error	103.47	12	8.62		

**Table 10:** Analysis of variance of the obtained DoE response surface models of the response glass transition temperature (*T*_g_), determined via a DMA of EMC2, including degrees of freedom (d*f*), ration of two mean squares (*F*-value), and the null hypothesis significance test of 0.05 (*p*-value).

Source	Sum of Squares	d*f*	Mean Square	*F*-Value	*p*-Value
Model	73.41	3	24.47	49.18	<0.0001
A—Temperature	70.08	1	70.08	140.86	<0.0001
B—Injection Speed	0.0533	1	0.0533	0.1072	0.7486
A^2^	3.28	1	3.28	6.58	0.0235
Pure Error	6.45	12	0.5379		

**Table 11:** Analysis of variance of the obtained DoE response surface models of the response storage modulus, determined via a dynamic mechanical analysis (DMA) of EMC1, including degrees of freedom (d*f*), ration of two mean squares (*F*-value), and the null hypothesis significance test of 0.05 (*p*-value).

Source	Sum of Squares	d*f*	Mean Square	*F*-Value	*p*-Value
Model	5.817 × 10^5^	3	1.939 × 10^5^	47.80	<0.0001
A—Temperature	2670.08	1	2670.08	0.6583	0.4318
B—Injection Speed	5.406 × 10^5^	1	5.406 × 10^5^	133.28	<0.0001
AB	38,420.08	1	38,420.08	9.47	0.0088
Pure Error	48,868.00	12	4072.33		

**Table 12:** Analysis of variance of the obtained DoE response surface models of the response storage modulus, determined via dynamic mechanical analysis (DMA) of EMC 2, including degrees of freedom (d*f*), ration of two mean squares (*F*-value), and the null hypothesis significance test of 0.05 (*p*-value).

Source	Sum of Squares	Df	Mean Square	*F*-Value	*p*-Value
Model	4.806 × 10^6^	2	2.403 × 10^6^	10.74	0.0015
A—Temperature	2.651 × 10^6^	1	2.651 × 10^6^	11.84	0.0040
B—Injection Speed	2.156 × 10^6^	1	2.156 × 10^6^	9.63	0.0078
Pure Error	2.790 × 10^6^	12	2.325 × 10^6^		

## Data Availability

The original contributions presented in the study are included in the article/Appendix A, further inquiries can be directed to the corresponding authors.
